# In-Depth Characterization of Stromal Cells within the Tumor Microenvironment Yields Novel Therapeutic Targets

**DOI:** 10.3390/cancers13061466

**Published:** 2021-03-23

**Authors:** Sebastian G. Walter, Sebastian Scheidt, Robert Nißler, Christopher Gaisendrees, Kourosh Zarghooni, Frank A. Schildberg

**Affiliations:** 1 Department of Cardiothoracic Surgery, University Hospital Cologne, 50937 Cologne, Germany; sebastianwalter01@gmail.com (S.G.W.); christopher.gaisendrees@uk-koeln.de (C.G.); 2 Clinic for Orthopedics and Trauma Surgery, University Hospital Bonn, 53127 Bonn, Germany; sebastian.scheidt@ukbonn.de; 3 Institute of Physical Chemistry, Göttingen University, 37077 Göttingen, Germany; rnissle@gwdg.de; 4 Department of Orthopaedic Surgery, University Hospital Cologne, 50931 Cologne, Germany; kourosh.zarghooni@uk-koeln.de

**Keywords:** cancer-associated fibroblasts, mesenchymal stromal cells, fibroblast, cancer, tumor, tumor microenvironment, crosstalk

## Abstract

**Simple Summary:**

This up-to-date and in-depth review describes fibroblast-derived cells and their role within the tumor microenvironment for tumor progression. Moreover, targets for future antitumor therapies are summarized and potential aspects for future translational research are outlined. Furthermore, this review discusses the challenges and possible obstacles related to certain treatment targets.

**Abstract:**

Cells within the tumor stroma are essential for tumor progression. In particular, cancer-associated fibroblasts (CAF) and CAF precursor cells (resident fibroblasts and mesenchymal stromal cells) are responsible for the formation of the extracellular matrix in tumor tissue. Consequently, CAFs directly and indirectly mediate inflammation, metastasis, immunomodulation, angiogenesis, and the development of tumor chemoresistance, which is orchestrated by complex intercellular cytokine-mediated crosstalk. CAFs represent a strategic target in antitumor therapy but their heterogeneity hinders effective treatment regimes. In-depth understanding of CAF subpopulations and knowledge of specific functions in tumor progression will ultimately result in more specific and effective cancer treatments. This review provides a detailed description of CAFs and CAF precursor cells and summarizes possible treatment strategies as well as molecular targets of these cells in antitumor therapies.

## 1. Introduction

Tumors are “fibrotic wounds that do not heal” [1]. The fibrosis-like tissue of desmoplastic tumors is stiffer than the surrounding healthy tissue and is primarily detected by physical palpation and radiologic imaging; nonetheless, the molecular significance of this stiffness for tumor progression has remained unclear for generations [2].

**Figure 1:**
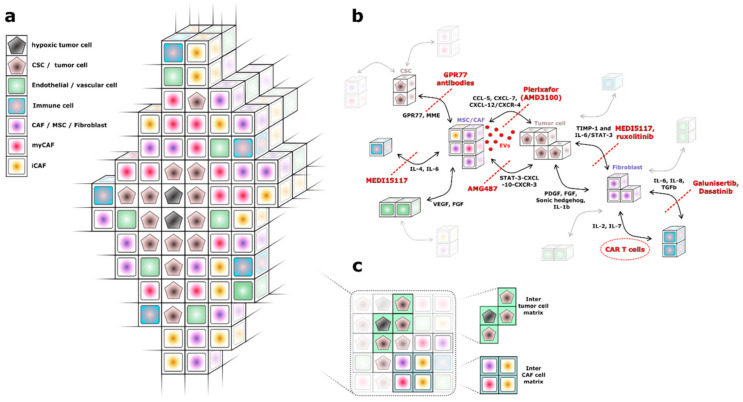
(**a**) Model of tumor cells and cells of the tumor microenvironment (TME). The TME contains multiple cell types of diverse origins (endothelial, immune, fibroblastic) but the location of specific subtypes is characteristic of certain cancer-associated fibroblast (CAF) subtypes (myofibroblastic CAFs (myCAFs), adjacent to tumor, and “inflammatory” CAFs (iCAFs), in the TME periphery). (**b**) Complex crosstalk between different cells (tumor cells and TME cells) yields several specific targets for the inhibition of tumor growth by specific molecules. However, several intercellular interactions of TME cells have not yet been studied in sufficient detail to establish an adequate target strategy (e.g., immune cells and vascular cells). (**c**) The extracellular matrix is depicted in this subfigure. The matrix accounts for a large part of the TME and represents a potential therapeutic target.

Tumors consist of a tumor bed (parenchyma), which describes the compartment of tumor cells and cancer stem cells, and a tumor microenvironment (TME). At first glance, the TME appears as a chaotic and disorganized structure. From a histological perspective, the TME consists of: (a) immune cells such as tumor-associated macrophages (TAM), natural killer (NK) cells, neutrophils, mast cells, dendritic cells (DC), CD4+ and CD8+ T cells, and B cells; (b) vascular cells including microvascular cells, endothelial cells (EC), and pericytes; (c) extracellular matrix molecules including collagen, glycoproteins, and proteoglycans; and (d) nonmalignant cells of mesenchymal origin such as fibroblasts, mesenchymal stromal cells (MSC), and cancer-associated fibroblasts (CAF) [3] (Figure 1a). The stroma is part of the TME and consists of a complex intercellular interstitium (matrix), expressed by the aforementioned cells of different function and origin that are located in-between. In recent years, there has been rapidly emerging evidence that the interaction between the matrix, tumor-associated cells, and tumor cells is essential for rapid tumor growth, limited responsiveness to therapeutics, and metastasis-initiating cells [1,4,5].

In the past, cancer stem cells (CSC) were the primary focus in oncologic research and multiple molecular targets were identified. Subsequent clinical trials, however, failed to stop tumor progression, provoked chemo-resistance, and accelerated tumor growth. In this context, inhibiting focal adhesion kinase (FAK) by defactinib, or STAT-3 by napabucasin, or, more prominently, treatment with the anti-DLL-3 antibody-drug conjugate rovalpituzumab tesirine (Rova-T) did not reach the expected clinical activity and efficacy [6,7,8]. It has to be postulated that the failed clinical therapeutic success of CSC targeting strategies is due to the CSC niche [9]. The latter describes an anatomically distinct region within the tumor microenvironment that maintains the principal properties of CSCs, preserves their phenotypic plasticity, protects them from the immune system, and facilitates their metastatic potential [9]. Several studies identified this niche as a promising target for anticancer therapy; however, these aspects are beyond the purpose of this review and were reviewed elsewhere [10,11,12,13].

Research continued to investigate cells within the stroma of desmoplastic tumors as possible treatment targets. Major findings were that cell activities within the TME are similar to those in chronically inflamed tissues and that intercellular crosstalk between stromal cells and CSCs is essential for tumor progression. In nontumor tissue, wound repair is initiated by infiltration of inflammatory cells that secrete growth factors, chemo- and cytokines, as well as matrix metalloproteinases (MMPs) [14,15]. Bone marrow-derived fibroblasts are recruited for remodeling of fibrin clots and deposition of extracellular matrix (ECM) proteins [16]. Through deposition of these ECM proteins, a three-dimensional network is established that enables fibroblasts to differentiate into myofibroblasts through mechanical tension [17].

In the case of tumors, fibrotic tissue is characterized by chronic inflammation and an increased density of myofibroblasts that build an extremely dense and rigid ECM network [18]. Cytokines expressed by stromal cells enable crosstalk between different cell types in tumor tissue, further enhancing tumor promotion and restructuring the stroma for malignant transition [19,20,21]. Restructuring of the stroma is closely associated with matrix protein deposition that is different from that of healthy stromal tissue and includes secretion of tenascin, periostin, SPARC, and collagens [22,23,24,25]. The altered biochemical properties and irregular anatomy of the ECM result in tissue stiffening, extremely elevated cytoskeletal tension, and fibrosis, which present an ideal niche for malignant transformation, tumor progression, and immune-evasion [26,27,28]. Matrix stiffening is promoted by the TGFβ-stimulated production of collagen and the collagen crosslinking enzyme lysyl oxidase (LOX) [29]. Once the tumor stroma presents hallmarks such as an increased ECM density; presence of CAFs; abnormal, disorganized, or leaky vascularization; and infiltration by innate and adaptive immune cells with pro- and antitumor activity, the underlying tumor is of increased aggressiveness and metastatic potential [30]. In this context, the stroma has been identified as important and unneglectable co-target in anticancer therapy [31,32,33].

Recent studies and the emerging focus on cancer biology have sought to develop a more profound and mechanistic understanding of the role of the TME in the progression of malignant tumors, and large-scale genomic analysis, single cell RNA-sequencing, and metabolomic data have identified a variety of stromal cell subtypes within the TME [3].

This review describes the role of fibroblast-derived cells and their role within the TME for tumor progression. Moreover, targets for future antitumor therapies are summarized and potential aspects for future translational research are outlined. Furthermore, this review discusses challenges and possible obstacles of certain treatment targets.

## 2. Tumor Stromal Cells of Mesenchymal Origin

### 2.1. Resident Fibroblasts and Myofibroblasts

Fibroblasts are resident cells responsible for the synthesis, deposition, and structure of the ECM. Typically, fibroblasts are characterized by the presence of fibroblast activation protein (FAP). Deposition of ECM proteins such as collagen type I, type IV, proteoglycan, and fibronectin stimulates mechanoreciprocity, which is a term that defines the actomyosin cytoskeletal response to mechanical stress of the ECM. Transdifferentiation, which describes the de novo expression of α smooth muscle actin (αSMA) and the differentiation of fibroblasts into myofibroblasts, is also promoted by ECM deposition [34]. Upon the binding of transforming growth factor (TGF)-β1, fibroblasts increase expression of fibronectin, which subsequently results in increased mechanical tension through the binding of fibronexus adhesions, leading to the assembly of focal adhesions and αSMA recruitment to actomyosin fibers [34,35,36]. In contrast to short contractions of smooth muscle cells, αSMA-positive myofibroblasts are able to contract and thus stiffen the ECM permanently [37], which is mediated by the calcium-calmodulin-MLC kinase and Rho-ROCK-myosin light chain phosphatase [38]. Stiffened ECM enforces TGF-β1 release and thus further amplifies the activation of fibroblasts through a feedforward circuit, which is interrupted in healthy tissue by the activation of YAP/TAZ and MRTF [39,40,41]. These transmembrane proteins transduce mechanical stress to gene transcription and would ultimately lead to apoptosis or de-differentiation of myofibroblasts to quiescent fibroblasts [42]. In tumor tissue, the latter functions are disabled by the conversion of fibroblasts into CAFs through tumor cell-secreted platelet-derived growth factor (PDGF), fibroblast growth factor (FGF), sonic hedgehog (SHH), and IL-1β [43,44,45]. Another mechanism of fibroblast conversion to CAFs is induced by tissue inhibitor of metalloproteinase-1 (TIMP-1) and the subsequent activation of the IL-6/STAT3 pathway [46].

In vivo, vaccine-induced depletion of FAP-positive cells resulted in reduced tumor growth and less metastatic dissemination [47]. In this context, the predominant T helper cell (T_H_) phenotype changed from T_H_2 to T_H_1 concomitantly expressing more IL-2 and IL-7 instead of IL-6 or IL-4 [48]. Thus, more CD8+ T cells invaded the tumor tissue and displayed their cytolytic capacities.

### 2.2. Mesenchymal Stromal Cells (MSCs)

#### 2.2.1. Characterization and Recruitment

MSCs are multipotent cells that contribute to the regeneration of osseous, cartilaginous, fatty muscle tissue in case of chronic inflammation and participate in the immune response [49]. MSCs have been commonly defined by the presence of certain markers such as CD73, CD90, CD105, and the absence of CD45, CD34, CD14 or CD11b, CD79α or CD19, and HLA-DR [50], but emerging evidence suggests that these parameters are insufficient to define and subgroup the various phenotypes of this cell type [51,52].

In contrast to fibroblasts, MSCs are recruited and mobilized from the blood to the site of tissue injury by chemotaxis through growth factors such as TGF-β, PDGF, IGF, and FGF [53,54]; chemokines like CXCL-12 [55]; CCL25 signaling [56]; IL-6 [57]; and complement component 1 subcomponent q (C1q), C3a, and C5a [58,59]. There is evidence that MSCs (and other bone marrow-derived CD45+ myeloid cells) are attracted to tumor sites by similar signaling patterns in animal models [60,61]. Although it is likely that this phenomenon occurs in man as well, scientific data are still lacking. In wounded tissue, MSCs promote tissue repair by differentiating into activated fibroblasts, resident-like tissue stromal cells, or by directly mediating the activity of inflammatory cells at the site of the tissue lesion. For the latter, MSCs express and secrete vascular endothelial growth factor (VEGF), PDGF, and subsequently activate endothelial cells and prolong the survival of fibroblasts. Furthermore, MSCs promote immunomodulation by secreting IL-6, IL-8, and TGF-β [62,63]. Secretion of macrophage colony-stimulating factor (M-CSF) by MSCs increases the metabolization of cell debris by macrophages [64]. The regenerative function of MSCs can be hijacked by malignant tumors, and a large proportion of MSCs will subsequently differentiate into CAFs [65]. Once MSCs have differentiated into CAFs, they have the ability to maintain CSCs through the secretion of Notch ligand Jagged-1 [66].

In tumors, MSCs are responsible for immune-suppressive cues that inhibit T cell responses by secreting prostaglandin E2 (PGE_2_), TGF-β, and NO as well as expressing immunomodulatory enzymes such as IDO [67]. PGE_2_-release from MSCs is triggered via IL-1 signaling from carcinoma cells and subsequently induces expression of IL-6 and IL-8 [68].

#### 2.2.2. Tumor Promotion

In tumor tissue, MSCs seem to enhance cancer metastasis and tumor angiogenesis by secreting VEGF and β-fibroblast growth factor (FGF) [69] (Figure 1b).

It has been shown that breast cancer cells stimulate the MSCs to secrete CCL-5, which enhances motility, invasion, and metastasis of cancer cells through its paracrine function [70]. Most likely, CCL-5 expression stimulates PD-L1 expression in tumor cells [71].

MSC cytokine loops including IL-6 and CXCL-7 regulate CSCs and accelerate tumor growth [72]. MSCs are involved in development of chemoresistance of tumors. For instance, the number of MSCs significantly increased in a PDAC mouse model when exposed to gemcitabine, which was correlated to activation of the STAT-3-CXCL-10-CXCR-3 paracrine signaling axis and consequently promoted CSC survival [73]. However, inhibition of CXCL-10 by AMG487 resulted in a reduction of CSCs and enhanced gemcitabine efficacy [74].

In vitro exposure of HNSCC cells to paclitaxel resulted in the development of chemoresistance and consequently in increased survival of tumor cells when co-cultured with BMSC [75]. MSCs are also capable of inducing thermotolerance in tumor cells via the CXCL12 pathway, which may limit the effectiveness of HIPEC therapy [76].

Following a cisplatin treatment, MSCs increased expression of specific polyunsaturated fatty acids which promoted regrowth and multiplication of cancer cells [77].

However, there is evidence that the role of MSCs in tumor progression is ambiguous. In fact, MSCs hinder tumor progression by downregulating Akt activation respective signaling as has been observed in the context of Kaposi’s sarcoma and HCC [78,79]. Hepatoma cell proliferation was suppressed by the downregulation of nuclear factor-κB (NF-κB) expression through culturing within conditioned media of MSCs [80]. There is some evidence that MSCs have the potential to act as tumor suppressors via the Notch1 [52] and Wnt signaling pathway [53] or secreting exosomes or microvesicles [81]. In this context, tumor HepG2 cells incorporated MSC-released microvesicles and, subsequently, proliferative activity was significantly reduced, and apoptosis was induced. In analogy, adipose-derived mesenchymal stromal cell (ADMSC)-derived exosomes reduced tumor volume in a HCC rat model [82], and MHCC97-H human HCC cells had reduced invasive and metastatic potential after exposure and co-culturing with MSCs, which was contributed to stromal differentiation [83]. A recently identified antitumor effect of BMSCs is the downregulation of the PI3K/AKT signaling pathway [84].

These features expose MSCs as potential carriers for tumor-targeted therapies, e.g., by releasing extracellular vesicles at the tumor site [85,86]. Determining the precise role of signaling molecules such as autocrine motility factor (AMF), integrin-αvβ3, and microvesicle release triggers remains a task for further research [87,88].

### 2.3. Cancer-Associated Fibroblasts (CAFs)

#### 2.3.1. Definition and Interaction within ECM

Differentiated fibroblasts of the TME are collectively called CAFs and their density correlates with tumor aggression, metastasis potential, and patient survival in multiple tumor entities [89,90]. CAFs can be localized outside the TME in the case of metastasis. Although CAFs are heterogeneous, most are αSMA-positive [91]. They are proliferative, show high metabolic activity, and are depleted of FAP. They can be distinguished from myofibroblasts by their inability to undergo apoptosis or to de-differentiate into resting fibroblasts [30].

CAFs are recruited to the tumor side by similar cytokine-signaling mechanisms that recruit fibroblasts to wounds, and initially, CAFs may physically hinder tumor cells from invading surrounding tissue [92] as they may account for a large percentage of the total tumor volume [93]. However, as the tumor evolves, CAFs continue to deposit ECM proteins, secrete growth factors, and contract and remodel the ECM. As a consequence, CAFs re-organize and crosslink collagen to induce stiff and oriented collagen fibers along which tumor cells can migrate [30,94]. It is important to note that CAFs and not fibroblasts or myofibroblasts promote tumor progression by directing tumor cells away from the primary tumor, thus enabling metastasis [95,96]. Conversely, it seems that tumor cells are also capable of transforming fibroblasts into CAFs outside of the TME. For instance, metastasis of HCC-cells into lung tissue was promoted by HCC-released miR-1247-3p which transformed lung fibroblasts into CAFs with the capability of creating a niche for tumor cells [97].

#### 2.3.2. Interaction with Other Cell Types

CAFs are capable of promoting (chemo-)treatment resistance through a multitude of mechanisms that warrant further in-depth research; however, tumor promotion should not be associated with pro-stemness functions of CAFs alone [98]. CAFs promote tumor progression at an early stage of cancer development by the activation of NF-κB signaling through the release of IL-1 by immune cells [44]. CAFs are subsequently able to moderate immunologic response by influencing immune cell recruitment and activation at the tumor site and shifting the immune response in a pro-tumorigenic direction. For example, IL-4 and IL-6 secretion of CAFs accounts for the infiltration of tumor-associated macrophages (TAMs) and other immunosuppressive myeloid cells that promote immune-suppression [99]. Furthermore, IL-6 partially regulates the maintenance of the CSC phenotype through the STAT-3-NF-κB pathway [100,101]. CAF-release of IL-8 regulates a subtype of epithelial-like CSCs that maintain high aldehyde dehydrogenase (ALDH) activity and are highly proliferative and, thus, support the stemness properties of cancer cells (e.g., breast and PDAC) [102,103]. CAF-mediated release of IL-10 by tumor-associated macrophages (TAMs) decreases cytotoxic T cell activity within tumors and induces regulatory T cell responses [104,105]. Furthermore, MHC-I expression in tumor cells is downregulated and costimulatory molecule expression is suppressed [106]. This finally results in an immunosuppressive microenvironment around the tumor.

CAFs induce and recruit regulatory T cells (T_regs_) to repress antitumor immune responses and support tumor progression by the induction of CD4+ helper T (T_H_) lymphocytes to strengthen pro-tumorigenic T_H_2 and T_H_17 phenotypes [107,108]. Polarization of T_H_2 cells in tumors is promoted by thymic stromal lymphopoietin (TSLP) and is associated with a worse prognosis in patients [109].

In this context, MSCs were engineered to release IL-7 and IL-12 in order to promote T_H_1 polarization. Upon release of IFN-γ and TNFα by CAR T cells, MSCs polarized T_H_2 cells into a T_H_17/T_H_1 phenotype, subsequently releasing IL-2 and IL-15 and further activating CAR T cells [110].

CAFs are also able to suppress CD8+ cytotoxic T cells and NK cells by expressing programmed death-ligand (PD-L)1 and PD-L2 and can secrete immune suppressive factors such as PGE_2_ and IDO [111,112]. Stiffened and densified TME, as a result of elevated ECM deposition, further limits immune cell infiltration to the tumor.

Chemoresistance is triggered by CAFs as shown in human prostate cancer using the genotoxic agent mitoxantrone [32]. This agent apparently stimulates Wnt-16B secretion by CAFs and, thus, induces increased proliferation and invasion of carcinoma cells. In human colorectal cancer (CRC), chemotherapy-induced IL-17A production by CAFs promoted CSC self-renewal and tumor growth [113]. Chemoresistance of tumors can also be triggered by CAFs expressing G protein-coupled C5a receptor 77 (GPR77) and membrane metallo-endopeptidase (MME) that establish a survival niche for CSC [114].

#### 2.3.3. CAF Subtypes

Although most CAFs are αSMA-positive, there are CAF subgroups that do not express αSMA and co-exist with other CAF subtypes in the TME. Recent studies have underlined the hypothesis of tumor-promoting and tumor-restricting CAF subtypes [115,116]. However, to date there is no consensus on the molecular definition of CAFs (Table 1) [117].

Notably, CAF subtypes with a low expression of β1-integrin, FAP, and PDGF receptor (PDGFR)β were assigned to a luminal location and correlated with reduced tumor progressiveness. CAFs with a high expression of FAP, αSMA, and PDGFRβ were found to have immunosuppressive properties and are thus presumably associated with more aggressive breast cancer phenotypes, such as triple-negative cancers [115]. In analogy to breast cancer, CAFs in PDAC could be grouped into CD10-positive and -negative. CD10-positivity correlated with tumor progression and chemoresistance, through persistent NF-κB activation and resultant IL-6 and IL-8 secretion [114,118]. The aforementioned study by Su and colleagues identified that CD10+ and GPR77+ CAFs are a promising antitumor target as this CAF subpopulation establishes a survival niche for CSC by protecting them from chemotherapeutic attacks through ABCG2 expression in cancer cells [114].

Furthermore, CAF subsets seem to cluster at certain locations within the TME relative to the tumor. For instance, αSMA-expressing and FAP-positive myofibroblastic CAFs (myCAF) were adjacent to the tumor, while “inflammatory” CAFs (iCAF) with reduced αSMA expression were found in the dense stroma and secreted IL-6, CXCL-1, and CXCL-2 through the activation of IL-1α-Janus kinase (JAK)-STAT signaling [116,119]. αSMA expression seems to be an unprecise marker of tumor progression as expression differs among different CAF subtypes that correlate with tumor progressiveness [115,119].

Tumor release of IL-1 was shown to induce differentiation of CAFs into iCAFs and the binding of TGF-β1 promoted differentiation into myCAFs by blocking the IL-1/JAK/STAT pathway [116]. In squamous cell carcinoma, a subgroup of CAFs induced epithelial mesenchymal transition (EMT) of malignant keratinocytes through secretion of TGF-β and may thus be causal for the induction of stemness features in malignant cancer cells [120,121,122]. Other CAF markers are fibroblast specific protein (FSP)-1 and PDGFR-α/β and the well-regulated intercellular crosstalk between tumor cells and fibroblasts contributes to CAF subdifferentiation on a spatial and functional spectrum [43,123,124]. In this context, additional CAF subtypes were identified [125]: CAF-1 cells expressing FSP-1, which promote metastatic colonization of tumor cells through tenascin- and VEGF-mediated angiogenesis [126]; and CAF-2 cells that are characterized by the presence of αSMA, neural/glial antigen 2 (NG2), and PDGFRβ [127]. The CAF-2 cell type accounts for type I collagen deposition, forming the stiff ECM, which acts as a tissue barrier preventing antitumor cells (e.g., cytotoxic T lymphocytes) from infiltrating tumor tissue [128].

Other CAF subtypes are labelled CAF-N (normal) and CAF-D (divergent). While the former secrete hyaluronic acid and MMPs, the latter induce EMT by expressing TGF-β [120,132]. Other CAF subtypes have been identified in CRC and were named CAF-A and CAF-B; however, their specific function has not yet been identified [127]. In PDAC tumors, another subset of CAFs was identified: one group highly expressing meflin (a glycosylphosphatidylinositol-anchored protein), which was thus named meflin-rich CAF (rCAF); and another group poorly expressing meflin (pCAF). Interestingly, rCAF seemed to act as a tumor suppressor [131,133]. Most recently, Costa et al. identified four other CAF subtypes named CAF-S1 to CAF-S4 that expressed six markers in different ratios in breast cancer with different properties, and these results were reproduced in head, neck, and lung cancers [115,130,134]. Thus, CAF-S1 was strongly positive for all six markers and CAF-S2 was negative for all markers. CAF-S3 was defined by the expression of ITGB1, FSP-1, and PDGFRβ and CAF-S4 by the same markers and the additional expression of αSMA [115]. A subsequent study by the same group reasoned using transcriptomic data that the CAF-S1 type presents a spectrum of eight different functional clusters, each expressing specific genes coding ECM proteins (Table 2). Notably, different clusters are associated with different CAF types. For instance, clusters S0, 3, 4, 6, and 7 are associated with myCAFs and clusters S1, 2, and 5 are associated with iCAFs, especially IFN-γ-iCAFs.

Progress in identifying CAF subgroups with specific markers will result in the identification of novel potential and specific therapeutic targets. Furthermore, targeting CAFs, which account for a large percentage of the total tumor volume, may have better pharmacodynamics effects than focusing on CSCs only. From a histological point of view, CAFs are localized in the periphery of tumors and are thus directly accessible to therapeutic agents that diffuse from the blood, and breaking this outer wall might ultimately result in the breaching the cancer’s fortress [135].

## 3. Targeting Tumor Stroma Cells

Essentially, there are three potential treatment strategies when targeting stroma cells in cancer treatment: (1) using tumor-tropism of MSCs for the delivery of antitumor molecules; (2) directly targeting CAFs by depletion; and (3) inhibiting intercellular crosstalk between CAFs, tumor cells, and other cell types (Table 3).

As stated previously, fibroblasts and MSCs play a crucial role in tumor development and may therefore present a strategic target for anticancer therapies [86,98,136]. In healthy tissue, fibroblasts modulate immune cell reactions by excreting chemo- and cytokines with different specificities. For instance, in a mouse model for colon cancer inhibited tumor growth and metastatic dissemination, they targeted and killed FAP-positive cells via an oral DNA vaccine [47]. In this context, T cell distribution was altered as fewer T_H_2 cells but more T_H_1 cells were recruited, expressing IL-2 and IL-7, and infiltrated the tumor [48]. The authors concluded that a reduced infiltration of M2 macrophages resulted in reduced levels of type 2 cytokines and subsequently increased infiltration of CD8+ T cells, which enforce tumor-lytic activity. Accordingly, targeting CAR T cells against FAP increased endogenous antitumor immunity and thus presented an effective treatment strategy in the preclinical setting [137].

Other studies investigated the crosstalk between CAFs and CSCs. A study by Korkaya et al. attempted to inhibit the IL-6/STAT-3/NF-κB loop by functional blocking of IL-6 through an anti-IL-6 receptor antibody and found a decreased chemoresistance of HER2 breast cancer cells against trastuzumab [100].

A combination of different therapeutic agents for antitumor therapy at certain intervals may thus result in lower effective dosages. Low-dose metronomic (LDM) chemotherapy has become a clinically applicable strategy to enhance the success of antitumor therapy by converting the therapy-induced stromal alterations in desmoplastic cancers [138,139]. In the context of a mouse PDAC model, a low daily dosage of gemcitabine was additionally added to the high weekly dose, and in combination with Bv8 blockade of myeloid derived suppressor cells (MDSCs), a significant reduction in tumor growth, angiogenesis, and metastasis was reached [138]. A clinical study by Valenzuela demonstrated that patients with gastrointestinal tumors undergoing metronomic chemotherapy with a 5-fluorourcacil prodrug, celecoxib, and cyclophosphamide had predictive cytokine profiles that indicated treatment success. They also postulated that this drug combination impacted T_regs_ and suppressed MDSCs [139].

Inhibition of the nodal and activin receptor ALK4/7, which is expressed on pancreatic CSCs and stromal cells, using SB431542 reduced stemness markers and invasive capacities of CSCs in human pancreatic adenocarcinoma cell lines. Blockade of the ALK4/7 receptor in combination with gemcitabine led to decreased chemoresistance and subsequent complete CSC elimination [155]. Co-treatment with anti-PD-L1 and TGF-β blockers was associated with better tumor control [140].

Another strategy tested to achieve tumor remission was the inhibition of CD10+ and GPR77+ CAFs by IL-6 and IL-8 antibodies or anti-GPR-77 antibodies in combination with docetaxel in a xenograft model [114]. Other therapeutics targeting the IL-6-STAT-3 signaling axis are under development, including a high affinity anti-IL-6 antibody, MEDI5117, but they have not yet reached the clinical testing phase [141,142]. Another potential therapeutic agent inhibiting JAK is ruxolitinib, but its clinical relevance remains controversial [143,144]. As MSCs also express IL-6 and IL-8, these concepts synergize with MSC- and CAF-targeted therapeutics and effectively block the stroma-derived pro-stemness signals in desmoplastic tumors [68].

Increased expression of CC-chemokine-ligands (CCL) 2 to 5 by stromal cells attracts blood immature myeloid cells and T_regs_ to hypoxic tumor tissue where they initiate the expression of proangiogenic cytokines and enzymes or suppress the invasion of other immune cells, which promote tumor growth, angiogenesis, and metastasis [146,156]. CXC-chemokine ligand 12 (CXCL12) is another immune-modulating factor that is expressed by FAP+ CAF cells and is responsible for tumor cell survival and T cell compartmentalization within the TME in a lung cancer mouse model [147]. For antagonization of these effects, different small-molecule inhibitors of CXCR-2 such as AZ13381758 and SB225002 or AMD3100 (Plerixafor) in the case of CXCR-4 showed promising preclinical efficacy in PDAC models [31,145,157]. Blockade of the CXCL12 receptor CXCR4 resulted in limited tumor growth by rapid T-cell accumulation within the TME and subsequent sensibilization to checkpoint blockade therapy [147]. CXCL13 expression by FAP+ CAFS accounted for B and immunosuppressive plasma cell recruitment into the TME and is associated with clinical severity and malignant progression as demonstrated in a mouse model for prostate cancer [148].

Notably, the role of CXCL13 is ambiguous as CD8+ T lymphocytes are attracted and transcripts linked to cytotoxicity were increased in follicular T helper cells [158]. For instance, it was postulated that CXCL13-dependent immune cell recruitment to the TME creates a tumor mass in which durable antitumor responses can be generated [159].

Targeting of CAFs using a DNA vaccine directed on the FAP demonstrated tumor suppression and reduced the potential for metastasis in CRC and breast cancer models [47]. TGF-β presents a target for antitumor therapy as well its inhibition using SD208 or galunisertib (LY3200882) as the invasion of CD8+ T lymphocytes is increased and tumor growth is inhibited [149]. Furthermore, this resulted in reduced CAF expression of stemness markers and more differentiation markers in a CRC model [160]. A further therapeutic approach involves transducing CAFs with a nanocarrier-formulated plasmid encoding a secretable form of TNF-related apoptosis inducing ligand (TRAIL). As CAFs are intrinsically resistant to the effects of TRAIL, transduction transformed them into TRAIL-producing cells and triggered apoptosis of neighboring cancer cells [161]. Another recent therapeutic approach involves targeting of LIF: an IL family protein responsible for CSC promotion expressed by CAFs, which resulted in reducing the percentage of CSCs in a PDAC mouse model [150]. A more sophisticated approach involved CAR T cells that specifically killed FAP+ CAFs, delaying tumor growth in mice [151,152]. The combination of FAP+ CAF and EPH receptor A2 (EphA2)+ targeting resulted in complete tumor remission, which suggests a crucial supplemental role of CAF-targeting strategies along with conventional cancer therapies.

Nonetheless, depletion of CAFs does not necessarily result in tumor suppression as ganciclovir-induced depletion of CAFs resulted in the occurrence of invasive and undifferentiated tumors [153,162]. In this context, there is growing evidence to suggest that functional and unconventional inhibition of CAFs may be safer than their depletion. For example, vitamin D receptor (VDR) signaling has been shown to antagonize TGF-β/SMAD signaling-induced activation of stem cells in PDAC, which was initially mediated by IL-6, CCL-2, and CXCL-1 [154]. In combination with gemcitabine treatment, the vitamin D analog calcipotriol enhanced chemotherapeutic tumor control.

## 4. Conclusions

Heterogeneity in cellular composition represents a major challenge in the era of patient-specific modern oncology. In-depth understanding of intercellular crosstalk and the function of different cell types is crucial for the development of novel, cutting-edge antitumor therapies. Concomitant targeting of specific tumor stroma cells and especially CAFs will most certainly result in more effective antitumor protocols.

## Figures and Tables

**Table 1:** Summarizes different subtypes of CAFs that have been described by different studies. At this point, the interrelationship between different CAF subtypes and whether there are overlying phenotypes is not clear.

CAF Type	Markers and Proteins	Function	Specific Therapeutic Agents	Study
myCAF	αSMA, FAP, TGF-β, Collagen	metastasis, chemoresistance, proliferation	Losartan, Nab-paclitaxel, Galunisertib,	[116]
iCAF	IL-6, IL-8, CXCL-1, CXCL-2, LIF, CCL-2, CCL-17	inflammation, metastasis, angiogenesis, immunosuppression	Ruxolitinib	[116]
apCAF	MHCII, CD74+,	immunomodulation		[129]
CAF-1	FSP-1, VEGF, TNC	metastasis, angiogenesis	Dasatinib	[127]
CAF-2	αSMA, NG2, PDGFRβ, collagen	ECM formation, immunosuppression	Dasatinib	[127]
CAF-N	Hyaloronic acid, MMPs	ECM formation, Metastasis, immunosuppression	Losartan, Nab-paclitaxel, Galunisertib	[120]
CAF-D	TGF-β	invasion	Losartan, Nab-paclitaxel, Galunisertib	[120]
CAF-A	MMP2, DCN, COL1A2, FAP	ECM formation		[127]
CAF-B	ACTA2, TAGLN, PDGFA	ECM remodeling, metastasis		[127]
CAF-S1	FAP, TGF-β, CXCL-12, IL-6, IL-10, IL-17	metastasis, immunosuppression,	Dasatinib, Galunisertib	[130]
CAF-S2	FAP-, αSMA-, CD29-, PDGFRβ-	-to be investigated/ physiologic-		[130]
CAF-S3	FAP-, αSMA-, CD29-/+, PDGFRβ-/+	-to be investigated/ physiologic-	Dasatinib	[130]
CAF-S4	TGF-β, CXCL-12	metastasis, proliferation, angiogenesis	Dasatinib	[130]
pCAF	αSMA	proliferation, metastasis		[131]
rCAF	Meflin, αSMA-, PDGFRα, Gli1	tumor suppression		[131]

**Table 2:** CAF-S1 clusters that are described by the expression of different marker proteins and hence yield different tumor-modulating functions [134].

Cluster	Markers	Described Function
(0) ecm-myCAFs	LRRC15, GBJ2	Synergize with cluster 3
(1) detox-iCAFs	ADH1B, GPX3	
(2) IL-iCAFs	RGMA, SCARA5	
(3) TGFβ-myCAFs	CST1, TGFβ1	Upregulation PD-1 and CTLA4 in T_reg_
(4) wound-myCAFs	SEMA3C, SFRP4	Indicative for anti-PD-1 response
(5) IFNγ-iCAFs	CCL19, CCL5, CD74+	apCAF
(6) IFNαβ-myCAFs	IFIT3, IRF7	
(7) acto-myCAFs	GGH, PLP2	

**Table 3:** Overview of selected studies targeting stroma cells, indicating the type of study and the type of tumor studied.

Author	Year	Type of Study	Cancer Type	Short Summary
Sun Y. et al. [32]	2012	In vitro	prostate cancer	Damage to the tumor environment promotes prostate cancer therapy resistance
Loeffler M. et al. [47]	2006	In vitro/animal study (mice)	general	Tumor stromal antigen FAP can serve as a novel target for active vaccination against cancer
Liu C. et al. [72]	2011	In vitro	prostate cancer	MicroRNA miR-34a inhibits prostate cancer stem cells and metastasis by repressing CD44
Scherzed A. et al. [75]	2011	In vitro	HNSCC	Bone marrow derived stem cells enhance the survival of paclitaxel treated squamous cell carcinoma cells in vitro
Roodhart J. et al. [77]	2011	Animal (mice)	general	Mesenchymal stem cells induce resistance to chemotherapy through the release of platinum-induced fatty acids
Hombach A. et al. [110]	2020	In vitro	colorectal cancer	IL7-IL12 engineered mesenchymal stem cells improve A CAR T Cell attack against colorectal cancer cells
Lotti F. et al. [113]	2013	In vitro	colorectal cancer	Chemotherapy actives cancer-associated fibroblasts to maintain colorectal cancer-initiating cells by IL-17A
Jiao J. et al. [126]	2018	Animal study (mice)	hepatocellular carcinoma	Depletion of S100A4+ stromal cells reduces the stem cell-like phenotype of HCC but does not prevent tumor development
Mizutani Y. et al. [131]	2019	In vitro/animal study (mice)	pancreatic cancer	Meflin-positive cancer-associated fibroblasts inhibit pancreatic carinogenesis
Olive K.P. et al. [135]	2009	Animal study (mice)	pancreatic cancer	Inhibition of hedgehog signaling enhances delivery of chemotherapy in mice with pancreatic cancer
Wang L.C.S. et al. [137]	2014	In vitro/animal study (mice)	general	Targeting fibroblast activation protein with chimeric antigen receptor T cells can inhibit tumor growth
Hasnis E. et al. [138]	2014	In vitro/animal study (mice)	pancreatic cancer	Anti-Bv8 antibody and metronimic gemcitabine improve pancreatic andonocarcinoma
Valenzuela P. et al. [139]	2021	Clinical trial	gastrointestinal cancer	Multiplex cytokine measurements in GIT cancer patients
Mariathasan S. et al. [140]	2018	In vitro	general/urothelial cancer	TGFß attenuates tumor response to PD-L1 blockade by excluding T-cells
Zhong H. et al. [141]	2016	In vitro	general	IL-6 antibody sensitizes multiple tumor types to chemotherapy
Finkel K. A. et al. [142]	2016	In vitro	HNSCC	IL6 inhibition with MEDI5117 decreases the fraction of head and neck cancer stem cells
Park J.S. et al [143]	2019	Clinical trial	NSCLC	combination of afatinib and ruxolitinib in EGFR mutant NSCLC with progression on EGFR-TKIs
Hurwitz H.I. et al. [144]	2015	Clinical trial	pancreatic cancer	Roxolitinib or placebo in combination with capecitabine in patients with metastatic pancreatic cancer
Steele C.W. et al. [145]	2016	In vitro	pancreatic cancer	CXCR2 inhibition suppresses metastases and augments immunotherapy in pancreatic ductal adenocarcinoma
Tan W. et al. [146]	2011	In vitro	mammary cancer	Tumor-infiltrating T-cells stimulate mammary cancer metastasis through RANKL-RANK signaling
Feig C. et al. [147]	2013	In vitro/animal study (mice)	pancreatic cancer	Targeting CXCL12 from FAP synergizes with anti-PD-L1 immunotherapy in pancreatic cancer
Ammirante M. et al. [148]	2014	In vitro/animal study (mice)	prostate cancer	Tissue injury and hypoxia promote malignant progression of prostate cancer by inducing CXCL13 in tumor myofibrolasts
Holmgaard R.B. [149]	2018	In vitro/animal study (mice)	general	Targeting TGFß with galunisertib promotes antitumor immunity
Shi Y. et al. [150]	2019	In vitro/animal study (mice)	pancreatic cancer	Targeting LIF-mediated paracrine interaction in pancreatic cancer therapy
Kakarla S. et al. [151]	2013	In vitro	general	Antitumor effects of chimeric receptor engineered human T cells
Lo A. et al. [152]	2015	In vitro	general	Tumor-desmoplasia is disrupted by depleting FAP cells
Özdemir B. C. [153]	2014	In vitro/animal study (mice)	pancreatic cancer	Depletion of myofibroblasts in mouse pancreas cancer led to invasive tumors
Sherman M.H. [154]	2014	In vitro	pancreatic cancer	Vitamin D receptor stromal reprogramming suppresses pancreatitis and enhances pancreatic cancer therapy
